# Fatal Garlic (*Allium sativum*) Toxicosis in a Dog: Gross and Histopathological Findings in a Rare Case of Systemic Hemolytic Injury

**DOI:** 10.3390/ani16111712

**Published:** 2026-06-03

**Authors:** Elena Biasibetti, Valentina Maza, Virginia Tagliati, Simona Zoppi, Alessia Di Blasio, Elena Bozzetta, Marzia Pezzolato

**Affiliations:** Istituto Zooprofilattico Sperimentale del Piemonte, Liguria e Valle d’Aosta Via Bologna 148, 10154 Torino, Italy; elena.biasibetti@izsplv.it (E.B.); valentina.maza@izsplv.it (V.M.); virginia.tagliati@izsplv.it (V.T.); simona.zoppi@izsplv.it (S.Z.); elena.bozzetta@izsplv.it (E.B.); marzia.pezzolato@izsplv.it (M.P.)

**Keywords:** *Allium* toxicosis, oxidative hemolysis, canine poisoning, organosulfur compounds, methemoglobinemia, Heinz body anemia, veterinary pathology, post-mortem examination

## Abstract

Garlic (*Allium sativum*) contains organosulfur compounds capable of inducing oxidative damage to erythrocytes in dogs, leading to hemolytic anemia and methemoglobinemia. Although *Allium* toxicosis is relatively common, fatal cases with detailed histopathological characterization are rarely reported. This case report describes a fatal episode of garlic toxicosis in a 3-year-old female mixed-breed dog. At necropsy, gastric contents included food material and raw garlic cloves (16 g). Gross findings included pulmonary hyperemia and thoracic hemorrhagic effusion. Histopathological examination revealed multifocal myocardial hemorrhages, diffuse pulmonary capillary congestion, hepatic sinusoidal dilation with hemorrhagic areas, splenic hemorrhage, and renal glomerular capillary dilation. These lesions were consistent with systemic hypoxic and vascular injury secondary to oxidative erythrocyte damage. The lower quantity of garlic recovered compared with reported toxic doses suggests possible individual susceptibility or incomplete recovery of ingested material. This case highlights the diagnostic value of histopathology in suspected *Allium* fatal toxicosis and contributes to increasing the limited literature describing these outcomes in dogs.

## Simple Summary

Garlic (*Allium sativum*) is widely used in human nutrition and is often perceived as beneficial. However, ingestion of garlic may cause severe toxicosis in dogs due to oxidative damage to erythrocytes. Fatal cases are uncommon, and histopathological descriptions are limited. This report describes the gross and microscopic findings observed in a dog that died following garlic ingestion, highlighting the importance of histological examination in suspected cases of *Allium* toxicosis.

## 1. Introduction

Garlic (*Allium sativum*) is a cultivated plant traditionally classified within the Liliaceae family, widely used as a culinary ingredient. The toxic effects of garlic are mainly associated with organosulfur compounds, particularly allyl disulfides and sodium 2-propenyl thiosulfate, which exert oxidative damage on canine erythrocytes [1].

When garlic is crushed or chopped, allicin (diallyl thiosulfinate, diallyl disulfide) is also formed. It is responsible for many of garlic’s health benefits, including antibacterial, antiviral, and antifungal properties, antioxidant activity, cardiovascular effects, anticoagulant action, and anti-inflammatory properties [2]. The genus *Allium* also includes other plants widely used in cooking, such as onion (*Allium cepa*), leek (*Allium porrum*), chives (*Allium schoenoprasum*), and shallot (*Allium ascalonicum*). Like garlic, all these plants contain alkyl polysulfides.

Despite its beneficial properties in humans, the genus *Allium* is toxic to dogs, and ingestion can lead to severe consequences. Toxicity is mainly due to the sulfur-containing compounds present in these plants, which can damage erythrocytes in dogs, causing hemolytic anemia [3,4]. In particular, allyl disulfides present in *Allium sativum* wield a strong pro-oxidant effect on red blood cells, attributable to inhibition of the enzyme glucose-6-phosphate dehydrogenase (G6PDH). The resulting increase in reactive oxygen species in the bloodstream leads to erythrocyte lysis, Heinz body and eccentrocyte formation, and methemoglobin production. In association with hemolytic anemia, allicin and ajoene contained in garlic exert a muscle relaxant effect on the myocardium and vascular smooth muscle, resulting in hypotension [5,6]. Furthermore, ajoene and other organosulfur compounds exhibit antithrombotic activity [7]. These effects may therefore exacerbate the consequences of anemia and impaired oxygen delivery to tissues.

In dogs, the toxic dose is reported as 5 g/kg for *Allium sativum* (garlic) and 15–30 g/kg for *Allium cepa* (onion) [8]. Clinical signs of garlic toxicosis may appear within one day following ingestion in cases of large amounts, or several days after consumption. Clinically, toxicosis may manifest as depression, hemoglobinuria, jaundice, tachypnea, tachycardia, weakness, exercise intolerance, and increased susceptibility to cold [8]. At lower doses, clinical signs may be limited to abdominal pain and diarrhea, resulting from direct damage to the gastroenteric mucosa induced by garlic-based preparations [9].

Although cases of *Allium* toxicosis in domestic animals are not uncommon, they generally result in resolution of toxicosis and recovery of affected animals [10,11,12]. Fatal cases are rare, and histopathological descriptions of the organs involved in this toxicosis are even more scarce in the literature [13,14].

The aim of this report is to describe the gross and histopathological findings observed in a fatal case of garlic toxicosis in an adult dog.

## 2. Case Description

A 3-year-old female mongrel dog, weighing about 20 kg, was submitted for post-mortem examination to the Laboratory of the Istituto Zooprofilattico Sperimentale del Piemonte, Liguria e Valle d’Aosta (IZSPLV) in Turin. According to clinical history, the dog had shown gastrointestinal symptoms, including vomiting.

At necropsy, reddish and heavily congested oral mucosa were observed. Upon opening the carcass, food residues and two garlic cloves, with a total weight of about 16 g, were found in the stomach. The lungs appeared diffusely hyperemic, and hemorrhagic effusions were present within the thoracic cavity.

Additionally, a wooden skewer stick was found within the wrapping cloth surrounding the body, suggesting that the garlic may have been part of a prepared food item ingested by the animal. A complete examination of the gastrointestinal tract did not reveal perforations, ulcerations, peritonitis, or traumatic lesions attributable to the wooden skewer.

According to the panel in use at the IZPLV laboratories, toxicological analyses were performed on gastric contents and vomitus by liquid chromatography–mass spectrometry (LC-MS) and gas chromatography–mass spectrometry (GC–MS) resulting negative for anticoagulant rodenticides, organophosphates, carbamates, and metaldehyde.

Vital organs (heart, lungs, spleen, liver, and kidneys) were sampled for histological examination. Tissues were fixed in 10% buffered formalin, trimmed, routinely processed, paraffin-embedded, and sectioned at 3–5 µm thickness. All sections were stained with hematoxylin and eosin and examined microscopically using an Olympus BX60 light microscope (Olympus Corporation, Tokyo, Japan). Histological images were acquired using DP-Soft software version 2.1.

**Figure 1:**
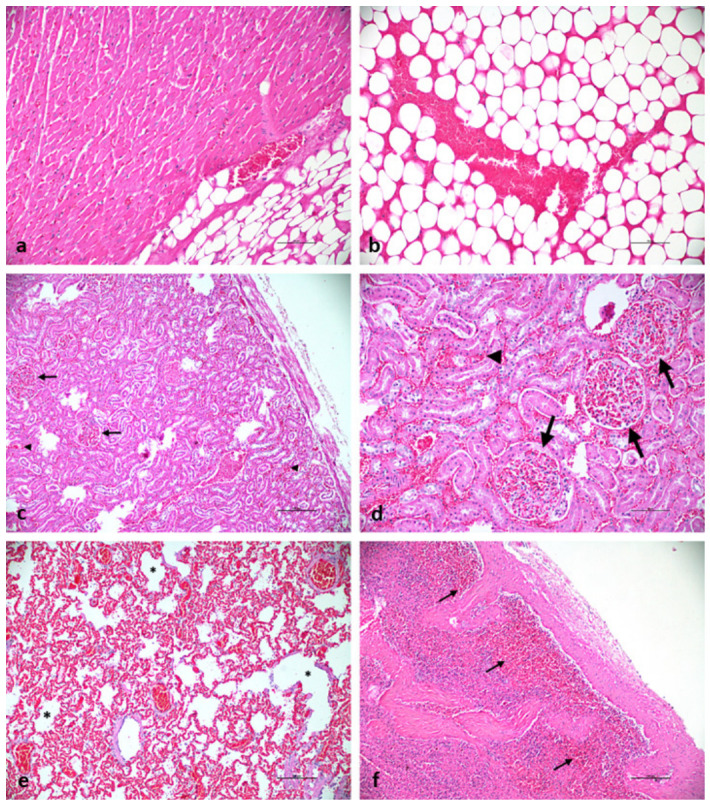
Histological lesions observed in the examined organs: (**a**) multifocal myocardial hemorrhages, 10× magnification, scale bar measuring 100 µm; (**b**) hemorrhagic area in the fat tissue of the heart, 10× magnification, scale bar measuring 100 µm; (**c**) glomerular capillary dilation (arrow) and tubular hemorrhages (arrowhead), 10× magnification, scale bar measuring 100 µm; (**d**) glomerular capillary dilation (arrow) and tubular hemorrhages (arrowhead), 20× magnification, scale bar measuring 50 µm; (**e**) alveolar capillary congestion and emphysema (asterisk), 10× magnification scale bar measuring 100 µm; (**f**) focal hemorrhages in the spleen parenchyma (arrow), 10× magnification, scale bar measuring 100 µm.

Histologically myocardium showed multifocal interstitial hemorrhagic areas. The pulmonary parenchyma showed marked and diffuse dilation of alveolar capillaries consistent with passive congestion, associated with focal areas of alveolar emphysema. The hepatic parenchyma exhibited diffuse blood congestion, with marked dilation of sinusoids and vessels and the presence of hemorrhagic areas. Focal hemorrhagic areas were also evident in the splenic parenchyma. In the kidneys, marked dilation of glomerular capillaries and capsular hemorrhagic foci were observed (Figure 1).

Overall, observed lesions were suggestive of systemic vascular congestion and hemorrhage compatible with severe hypoxic injury secondary to hemolytic toxicosis (Table 1). Macroscopic and microscopic findings were consistent with garlic toxicosis [13].

## 3. Discussion

According to the 2025 report of the Veterinary Poisons Information Service, toxicosis caused by plant species belonging to the genus *Allium* represents a frequent cause of poisoning in domestic animals in the UK, with a total of 1585 reports recorded in the year of publication [15]. Canine and feline poisoning cases following *Allium* ingestion were also reported in Spain and Netherlands [16]. These plants are widely used in cooking and agriculture and are therefore easily accessible to domestic animals. Moreover, considering the beneficial effects attributed to garlic in humans, some owners may improperly administer homemade preparations containing garlic or onion to their pets. However, this kind of toxicosis is usually resolved with support therapy which leads to the animal’s recovery [10,11,12].

As already stated, the compounds responsible for toxicity are organosulfur compounds released during mastication or cooking of the plants [17,18]. These compounds lead to reduced G6PDH activity and increased methemoglobin concentration within erythrocytes [19,20,21]. G6PDH plays a key role in regenerating reduced glutathione from oxidized glutathione; its inhibition therefore results in increased reactive oxygen species, responsible for hemoglobin denaturation and precipitation, leading to Heinz body formation [22,23]. Hemoglobin denaturation due to oxidative stress results in hemichrome formation. Hemichromes bind to the band 3 proteins on the red blood cells membrane, causing the membrane’s lipid bilayers to fuse together and collapse. The result of this process creates an “hemi-ghost” or “bite cell” appearance under a microscope. Red blood cells undergoing this process are termed “eccentrocytes” [18]. Direct oxidative damage to the erythrocyte membrane, together with Heinz body accumulation and eccentrocyte formation, ultimately results in erythrocyte lysis [24,25].

Clinical manifestations of toxicosis are thus attributable to the development of hemolytic anemia and increased circulating methemoglobin, with a consequent reduction in oxygen transport to tissues [8]. Additionally, fresh garlic-based preparations can damage the gastric and enteric mucosa, resulting in abdominal pain, vomiting episodes and diarrhea [9].

Clinical signs may also occur several days after ingestion. Initially, profuse vomiting and diarrhea, anorexia, and dehydration may be observed. Subsequently, hemolytic effects develop, with polypnea as a consequence of tissue hypoxia, initially pale mucosa due to erythrocyte damage, followed by icterus. Jaundice initially has a hemolytic origin and later becomes hepatotoxic, as the liver struggles with erythrocyte breakdown [26]. Hemoglobinuria and methemoglobinuria may also be observed [15].

The dog described in the present case report was submitted to necropsy after acute clinical deterioration characterized by gastrointestinal signs. The finding of a wooden skewer stick, together with the gross and histological findings, suggests that death was caused by ingestion of a lethal dose of garlic. Even if the garlic cloves recovered (about 16 g in weight) were lower than the toxic dose commonly reported in the literature (approximately 5 g/kg) in relation to the dogs’ weight, several factors could explain the alleged causal link between garlic consumption and death, including incomplete recovery of ingested material, comprehensive of entire intestinal content, during necropsy, prior digestion, cumulative exposure, previous diarrhea or individual susceptibility. Breed-related differences in erythrocyte metabolism and antioxidant capacity have been reported and may increase sensitivity to oxidative damage. Dogs with high levels of erythrocyte reduced glutathione and circulating potassium are more susceptible to the hematological effects of *Allium* species. This characteristic is relatively common in certain Japanese breeds, such as Akita Inu and Shiba Inu [27]. Other congenital metabolic defects or nutritional deficiencies that reduce erythrocyte antioxidant capacity, such as G6PDH or zinc deficiency, may further increase susceptibility to *Allium* toxicosis [28].

No hematological or biochemical data, including blood smears for Heinz body and/or eccentrocytes detection or methemoglobin measurements, were available because no blood samples had been collected prior to death and this represents a limitation of the present case. Furthermore, the gross and microscopic findings, together with the presence of garlic in the stomach and the exclusion of alternative toxic causes of death, support a diagnosis of fatal acute toxicosis due to accidental consumption of garlic.

## 4. Conclusions

This case report describes a rare fatal case of *Allium sativum* toxicosis in a dog, highlighting the meaningful contribution of histological examination in establishing the diagnostic picture. In the absence of specific chemical and toxicological analyses, the microscopic findings observed in multiple organs, predominantly characterized by diffuse vascular and hemorrhagic alterations, provided essential support for diagnostic confirmation.

The observation of systemic histopathological lesions in the absence of specific gross findings underscores the importance of histological investigation as a fundamental diagnostic tool in suspected cases of garlic toxicosis, particularly when it is not feasible to obtain the exact ingested dose of garlic. This case contributes to the limited available histopathological descriptions and reinforces the value of the anatomo-pathological approach in the study of *Allium* toxicoses in dogs.

## Figures and Tables

**Table 1:** Summary of macroscopic and microscopic lesions observed in the examined organs.

Organ	Gross Findings	Histopathological Findings	Pathophysiological Interpretation
Heart	Multifocal hemorrhagic areas	Multifocal myocardial hemorrhages	Hypoxic and vascular injury secondary to hemolytic anemia
Lungs	Diffuse hyperemia; thoracic hemorrhagic effusion	Marked alveolar capillary congestion; focal emphysema	Passive congestion and impaired oxygen exchange
Liver	Diffuse congestion	Sinusoidal dilation; multifocal hemorrhages	Reduced oxygen delivery; circulatory compromise
Spleen	No significant gross lesions	Focal hemorrhages	Vascular fragility and systemic hypoxia
Kidney	No significant gross lesions	Glomerular capillary dilation; capsular hemorrhage	Circulatory disturbance and vascular damage

## Data Availability

The original contributions presented in this study are included in the article. Further inquiries can be directed to the corresponding author.

## Abbreviations

The following abbreviations are used in this manuscript:

G6PDH	Glucose-6-Phosphate Dehydrogenase
IZSPLV	Istituto Zooprofilattico Sperimentale del Piemonte, Liguria e Valle d’Aosta
